# Traditional Chinese medicine for frailty and sarcopenia in aging populations: a bibliometric and visualized analysis of research trajectories and emerging frontiers

**DOI:** 10.3389/fragi.2026.1854710

**Published:** 2026-07-31

**Authors:** Xiaoshuo He, Lisha Sun, Huizhen Chen, Qiu Chen

**Affiliations:** 1 Hospital of Chengdu University of Traditional Chinese Medicine, Chengdu, Sichuan, China; 2 Chengdu University of Traditional Chinese Medicine, Chengdu, Sichuan, China; 3 King’s College London, London, United Kingdom

**Keywords:** aging, bibliometric analysis, CiteSpace, frailty, research trends, sarcopenia, traditional Chinese medicine, VOSviewer

## Abstract

**Background:**

By 2050, the proportion of people aged 65 and older is projected to reach 16% globally, up from roughly 10% in 2022. Frailty and sarcopenia are among the most clinically consequential age-related conditions, independently associated with falls, hospitalization, and death. Traditional Chinese medicine (TCM)—encompassing herbal formulas, acupuncture, tai chi, and qigong—has shown therapeutic potential for both conditions, yet no systematic bibliometric overview of this research area exists.

**Methods:**

Publications on TCM interventions for frailty and sarcopenia were retrieved from the Web of Science Core Collection on 14 March 2026. After a four-phase screening (identification, document-type screening, title/abstract relevance screening, and inclusion), 405 publications were retained. Bibliometric and visualization analyses were performed using CiteSpace (6.2.R6), VOSviewer (1.6.20), and the R package bibliometrix (4.3.0). To avoid tautological bias, all Boolean search terms were removed from the keyword field before every keyword-based analysis (co-occurrence, growth, burst detection, thematic mapping, and thematic evolution).

**Results:**

A total of 405 publications from 2011 to 2026 were included. Annual output grew sharply after 2019 and peaked at 77 publications in 2025. China was the overwhelmingly dominant contributor (910 author appearances), followed by the USA (152) and Japan (131). With search terms excluded, keyword co-occurrence resolved into interpretable clusters: exercise/physical activity/aging; randomized controlled trials of muscle strength and physical function; falls, balance and rehabilitation; evidence synthesis (systematic review/meta-analysis); and cachexia/nutrition/skeletal-muscle inflammation. Burst detection showed early activity in falls, balance, elderly, and exercise (2011–2018) shifting toward network pharmacology, molecular docking, ninjin’yoeito, and nutrition (2020–2026). Co-citation analysis identified the seminal frailty-phenotype study as the foundational reference, with a strong tai-chi fall-prevention trial base and the EWGSOP/AWGS sarcopenia consensus definitions as intellectual pillars.

**Conclusion:**

TCM research on frailty and sarcopenia has grown substantially, particularly since 2019, with China as the primary driver and Japan and the USA as secondary contributors. Network pharmacology, nutrition-based intervention science, and oxidative-stress/inflammation mechanisms represent the most promising emerging frontiers. These findings provide a structured evidence map for prioritizing future research and translational efforts in TCM-based geriatric care.

## Highlights

- First bibliometric analysis mapping TCM interventions for frailty and sarcopenia.
- Analysis of 405 publications from the Web of Science Core Collection (2011–2026), retained through a documented four-phase screening.
- Annual output accelerated after 2019 and peaked at 77 publications in 2025; China is the dominant contributor.
- With search terms excluded, exercise, falls/balance, cancer cachexia, and network pharmacology emerge as the core knowledge structure.
- Network pharmacology, nutrition, and oxidative-stress mechanisms are the fastest-emerging research frontiers.

## 1 Introduction

By 2050, more than 1.6 billion people will be aged 65 and older—roughly 16% of the global population, up from 10% in 2022 (United Nations et al., 2022). This demographic shift is driving a rise in age-related conditions that undermine independence and strain healthcare systems. Among these, frailty and sarcopenia stand out for their prevalence and their downstream consequences: falls, hospitalization, institutionalization, and death (Clegg et al., 2013; Cruz-Jentoft et al., 2019).

Frailty is a multidimensional clinical syndrome characterized by diminished physiological reserves and heightened vulnerability to stressors, resulting in disproportionate declines in health status following minor perturbations (Fried et al., 2001). The condition affects an estimated 12%–24% of community-dwelling older adults globally and is independently associated with increased risks of falls, hospitalization, institutionalization, and mortality (Collard et al., 2012; Kojima, 2015). Sarcopenia, defined as the age-related progressive loss of skeletal muscle mass, strength, and physical performance, shares substantial pathophysiological overlap with frailty and frequently co-occurs in what has been termed the “frailty–sarcopenia syndrome” (Cruz-Jentoft et al., 2019; Landi et al., 2015). The EWGSOP2 revised consensus placed muscle strength at the center of sarcopenia diagnosis, a refinement that has substantially shaped how researchers design intervention studies (Cruz-Jentoft et al., 2019).

Current evidence-based interventions for frailty and sarcopenia primarily encompass resistance exercise training, nutritional supplementation (particularly protein and vitamin D), and multicomponent intervention programs (Dent et al., 2019; Vlietstra et al., 2018). However, adherence to exercise regimens remains challenging in frail populations, pharmacological options are limited, and the heterogeneous nature of these syndromes necessitates individualized, multimodal therapeutic approaches. These gaps have driven interest in multimodal complementary approaches, TCM in particular, which combines physical, nutritional, and pharmacological components within a single therapeutic system.

TCM has been practiced for over two thousand years and encompasses herbal formulas, acupuncture, moxibustion, tai chi, qigong (baduanjin), tuina, and dietary interventions. Several are directly relevant to frailty and sarcopenia: randomized trials show that tai chi and baduanjin improve balance, muscle strength, and functional performance in older adults (Huang et al., 2022; Niu et al., 2022). Chinese herbal formulas such as Bu-Zhong-Yi-Qi decoction and Liu-Wei-Di-Huang pill have been investigated for their potential to modulate inflammatory pathways, mitochondrial function, and muscle protein synthesis (Zhang et al., 2024). Acupuncture has demonstrated efficacy in alleviating fatigue, improving appetite, and enhancing physical function in geriatric populations (Ma et al., 2023). From a TCM theoretical perspective, frailty and sarcopenia align closely with the concepts of “Xu Lao” (deficiency-taxation syndrome) and “Wei Zheng” (atrophy syndrome), rooted in deficiencies of spleen–kidney qi and essence, a framework that underpins most TCM intervention strategies in this area (Guo et al., 2022).

Bibliometric analysis applies quantitative methods to bibliographic metadata—citation networks, co-authorship patterns, keyword co-occurrence, burst detection—to map research fields objectively and reproducibly (Donthu et al., 2021; Aria and Cuccurullo, 2017). Tools such as CiteSpace, VOSviewer, and the R bibliometrix package make it practical to track how a field’s focus has shifted over time (Aria and Cuccurullo, 2017; Chen, 2006; van Eck and Waltman, 2010). Bibliometric methods have been widely adopted in biomedical fields and are particularly useful for rapidly growing interdisciplinary areas where the volume of literature makes narrative synthesis incomplete.

No bibliometric study has yet systematically mapped this intersection. Prior reviews have covered individual modalities—tai chi for sarcopenia, acupuncture for frailty—but none has taken a field-wide view spanning both conditions and all TCM approaches. Therefore, the present study aims to: (1) delineate the publication landscape and growth trajectory of TCM research for frailty and sarcopenia; (2) identify the most influential countries, institutions, authors, and journals; (3) reveal the thematic structure and temporal evolution of research hotspots through keyword and co-citation analyses; and (4) detect emerging frontiers through burst analysis, providing a structured evidence map to guide future research in TCM-based geriatric care.

## 2 Materials and methods

### 2.1 Data source and search strategy

Bibliographic data were retrieved from the Web of Science Core Collection (WoSCC), which is widely recognized as an authoritative and comprehensive citation-indexing platform for bibliometric analyses owing to its rigorous journal-selection criteria and, critically, its standardized and fully parsed cited-reference metadata—the prerequisite for co-citation, bibliographic-coupling, and burst analyses (Birkle et al., 2020). The search was conducted on 14 March 2026, using the Topic (TS) field, which searches titles, abstracts, author keywords, and Keywords Plus®. We note that the WoSCC does not index records with MeSH or any equivalent controlled vocabulary (MeSH is specific to MEDLINE/PubMed); a carefully constructed free-text strategy is therefore the appropriate retrieval mechanism in this environment. The strategy combined three conceptual blocks with Boolean operators:

*Block 1 (TCM interventions):* TS=(“traditional Chinese medicine” OR “Chinese herbal medicine” OR “Chinese medicine” OR “TCM” OR “herbal formula*” OR “Chinese herb*” OR “acupuncture” OR “moxibustion” OR “tai chi” OR “taichi” OR “tai ji” OR “qigong” OR “qi gong” OR “baduanjin” OR “tuina” OR “tui na” OR “acupoint” OR “acupressure” OR “cupping” OR “Chinese patent medicine” OR “Kampo” OR “herbal decoction” OR “Chinese materia medica”)

*Block 2 (Frailty):* TS=(“frailty” OR “frail” OR “frail elderly” OR “frailty syndrome” OR “prefrailty” OR “pre-frailty” OR “physical frailty” OR “cognitive frailty”)

*Block 3 (Sarcopenia):* TS=(“sarcopenia” OR “sarcopenic” OR “muscle wasting” OR “muscle atrophy” OR “muscle loss” OR “skeletal muscle decline” OR “lean mass loss” OR “myopenia” OR “muscle weakness” OR “low muscle mass”)

The final formula was Block 1 AND (Block 2 OR Block 3). No language or date restrictions were imposed. The search was deliberately recall-oriented (sensitive) to avoid omitting relevant TCM literature indexed under heterogeneous terminology; the resulting precision cost was addressed by the relevance-screening step described below. The search yielded an initial set of 485 records.

### 2.2 Screening and eligibility

**FIGURE 1:**
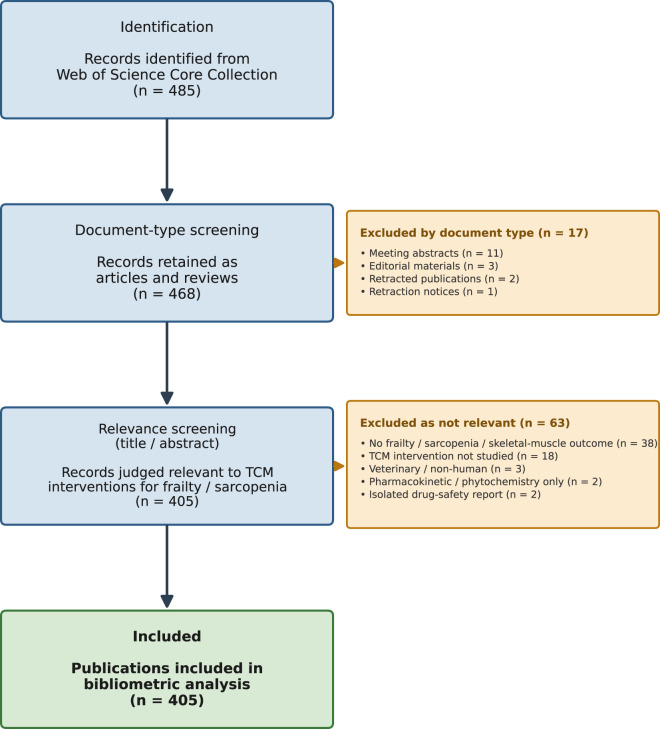
Flow diagram of record identification and screening (485 identified → 468 after document-type screening → 405 included).

Records were screened in a four-phase process (Figure 1).

1. Identification. The initial WoSCC search retrieved 485 records.
2. Document-type screening. Records were restricted to original articles and reviews. Seventeen records were excluded: 11 meeting abstracts, 3 editorial materials, 2 retracted publications, and 1 retraction notice, leaving 468 records.
3. Title/abstract relevance screening. Two authors independently screened the 468 records against pre-specified criteria; disagreements were resolved by discussion. A record was retained if it substantively investigated a TCM/mind-body intervention (or a TCM-derived agent) in relation to frailty, sarcopenia, skeletal-muscle mass/strength/function, muscle atrophy/wasting, or physical performance/balance/falls in a relevant population. A record was excluded when it had no genuine muscle/frailty/sarcopenia link, when the TCM intervention was not actually studied, when it reported only phytochemistry/pharmacokinetics or isolated drug-safety data, or when it was veterinary/non-human. This step removed 63 off-topic records (e.g., abbreviation collisions with “Transitional Care Model,” veterinary cases, and studies without a muscle/frailty outcome; full list with reasons in Supplementary Table S1).
4. Inclusion. 405 publications were retained for bibliometric analysis.

### 2.3 Analytical tools and parameters

Three complementary tools were employed. CiteSpace (6.2.R6; Drexel University, USA) was used for reference co-citation analysis, keyword burst detection, and temporal visualization (Chen, 2006); time slicing spanned 2011–2026 in 1-year intervals, with the top 50 items per slice (Top N = 50) and Pathfinder network pruning. VOSviewer (1.6.20; Leiden University, the Netherlands) was used for co-occurrence, co-authorship, and co-citation network construction (van Eck and Waltman, 2010), using association-strength normalization. The R package bibliometrix (4.3.0) on R 4.4.2 was used for descriptive analysis, three-field plots, thematic mapping, and thematic evolution (Aria and Cuccurullo, 2017).

In direct response to the requirement that retrieval terms must not contaminate the knowledge-structure analysis, all Boolean search terms from Blocks 1–3 (and their spelling variants) were removed from the author-keyword field prior to every keyword-based analysis (co-occurrence, keyword growth, thematic map, burst detection, thematic evolution, and the keyword axis of the three-field plot). The removed terms comprised the TCM-intervention, frailty, and sarcopenia terms listed in Section 2.1. Author keywords were used for co-occurrence, growth, and thematic analyses; a minimum occurrence threshold of 4 was applied to the co-occurrence network.

### 2.4 Ethical statement

As this study analyzed only publicly available bibliographic metadata and did not involve human subjects, animals, or primary data collection, ethical approval was not required.

### 2.5 Cross-database validation

To evaluate whether the findings were robust to the use of a single database, the equivalent search strategy was executed in Scopus (Elsevier) on the same topic fields and limited to articles and reviews. The Scopus and Web of Science records were harmonized and merged using the mergeDbSources() function of the bibliometrix package, with duplicates removed by DOI and title. We quantified the between-database document overlap and compared the leading countries, journals, and author keywords (search terms excluded) derived from Web of Science alone against those obtained from the merged corpus.

## 3 Results

### 3.1 General publication characteristics

**FIGURE 2:**
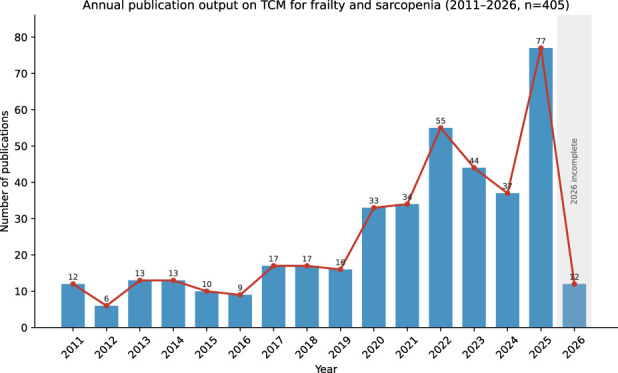
Annual publication output on TCM for frailty and sarcopenia, 2011–2026 (n = 405).

A total of 405 publications met the inclusion criteria. The dataset spanned 2011–2026, comprising work from 188 sources (journals) by 2,301 authors, with an average of 6.98 co-authors per document and 23.39 citations per document. Only 9 single-authored publications were identified, indicating a highly collaborative field. The international co-authorship rate was 17.78%, and the total number of cited references reached 18,758. Annual output remained modest from 2011 to 2016 (fewer than 15 publications per year), rose gradually during 2017–2019, and accelerated markedly after 2019—coinciding with the EWGSOP2 revised consensus on sarcopenia (Cruz-Jentoft et al., 2019) and growing global attention to aging. Output reached 55 publications in 2022 and peaked at 77 in 2025 (Figure 2). The lower count in 2026 reflects incomplete indexing, as the search was conducted in March 2026.

### 3.2 Countries/regions and institutional analysis

**FIGURE 3:**
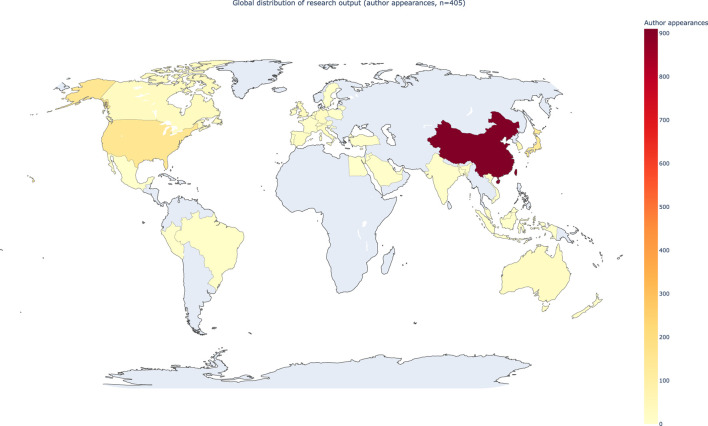
Global distribution of research output (all-author country appearances).

**FIGURE 4:**
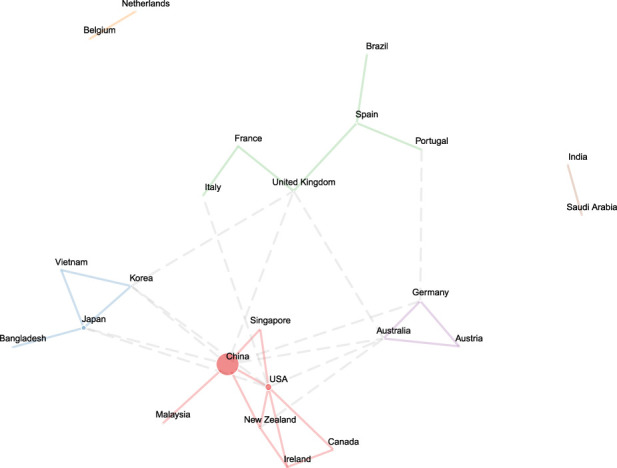
Country co-authorship network.

A total of 37 countries/regions contributed to the 405 publications. China was overwhelmingly dominant with 910 author appearances, followed by the USA (152), Japan (131), South Korea (44), the United Kingdom (33), Canada (31), Germany (27), Australia (26), Singapore (26), Italy (22), and Spain (22). By corresponding-author country, China again led (234 publications), followed by Japan (39) and the USA (33). The geographic distribution (Figure 3) shows a pronounced concentration of activity in East Asia, with secondary contributions from North America, Europe, and Oceania, and minimal engagement from Africa, the Middle East, and Central Asia. The country co-authorship network (Figure 4) shows China maintaining links with the USA, Japan, Australia, South Korea, and several European countries; the international co-authorship rate (17.78%) indicates that cross-national collaboration remains limited and represents an area for development.

### 3.3 Author and co-authorship analysis

**FIGURE 5:**
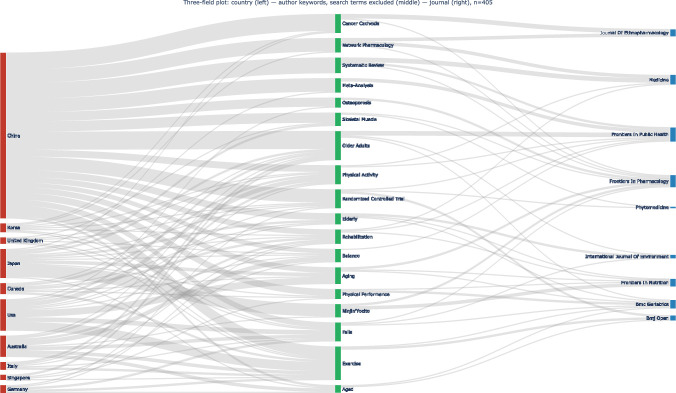
Three-field plot: country (left) — author keywords with search terms excluded (middle) — journal (right).

Among the 2,301 contributing authors, Zhang Y was the most prolific (12 publications), followed by Wang L and Wang Y (7 each), and by Guo HZ, Inui A, Klein JD, Li J, Li L, Li M, Li XY, Liu Y, Meng DY, Wang ZH, Yang G, and Zhang J (6 each). The three-field plot (Figure 5) shows distinct research orientations: Inui A concentrated on ninjin’yoeito (a Japanese Kampo formula) and sarcopenia; Guo HZ focused on TCM mechanisms of muscle atrophy; and Zhang Y showed the broadest scope across multiple keywords and journals. The predominance of Chinese-name authors is consistent with the geographic findings and reflects the strong foundation of TCM research in China.

### 3.4 Journal and co-citation analysis

**FIGURE 6:**
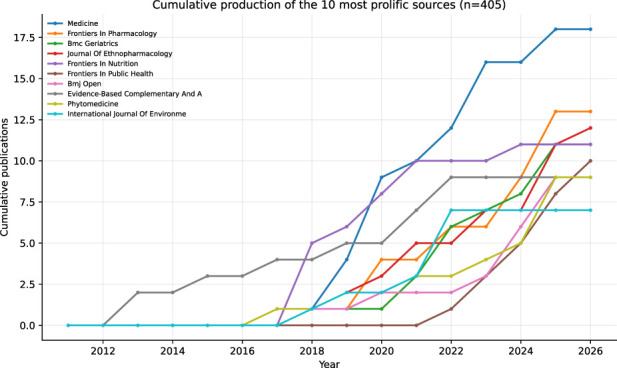
Cumulative production of the ten most prolific sources.

The 405 publications appeared in 188 sources, reflecting the interdisciplinary nature of the field. Medicine was the most prolific journal (18 publications), followed by Frontiers in Pharmacology (13), BMC Geriatrics and Journal of Ethnopharmacology (12 each), Frontiers in Nutrition (11), Frontiers in Public Health (10), BMJ Open, Evidence-Based Complementary and Alternative Medicine, and Phytomedicine (9 each), and the International Journal of Environmental Research and Public Health (7). The distribution spans geriatrics, pharmacology, nutrition, public health, and complementary medicine. Sources’ production over time (Figure 6) shows most journals entering the field after 2017, with Medicine demonstrating the most sustained cumulative growth.

### 3.5 Keyword co-occurrence and cluster analysis

**FIGURE 7:**
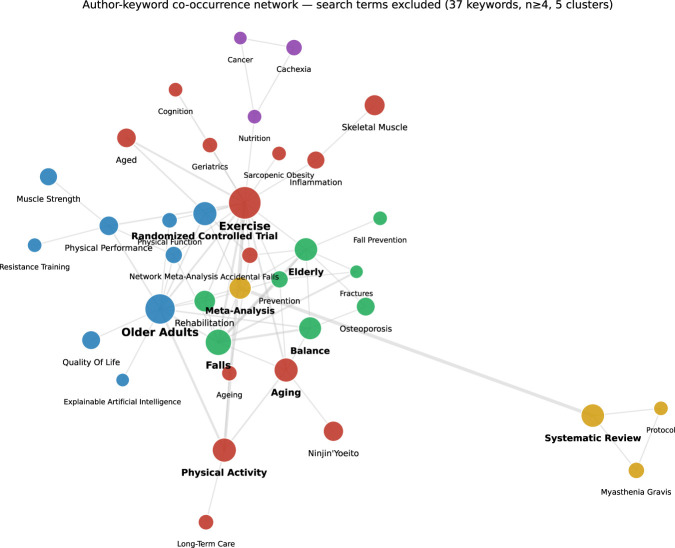
Author-keyword co-occurrence network with all search terms excluded.

After removing all search terms, the author-keyword co-occurrence network (VOSviewer; minimum occurrence = 4) organized 37 keywords into interpretable clusters (Figure 7): (i) an *exercise, physical activity and aging* cluster (exercise, physical activity, aging, aged, sarcopenic obesity, ninjin’yoeito); (ii) a *clinical-trial outcomes* cluster (older adults, randomized controlled trial, physical performance, muscle strength, physical function, quality of life, resistance training); (iii) a *falls, balance and rehabilitation* cluster (falls, elderly, balance, rehabilitation, osteoporosis, prevention, fall prevention); (iv) an *evidence-synthesis* cluster (systematic review, meta-analysis, protocol); and (v) a *cachexia/nutrition and skeletal-muscle mechanisms* group (cachexia, nutrition, skeletal muscle, inflammation). “Exercise,” “older adults,” and “falls” served as high-degree bridging nodes linking the clinical and mechanistic streams. Notably, once the retrieval terms were removed, the map reflects substantive research content rather than the search logic.

### 3.6 Keyword burst detection and emerging trends

**FIGURE 8:**
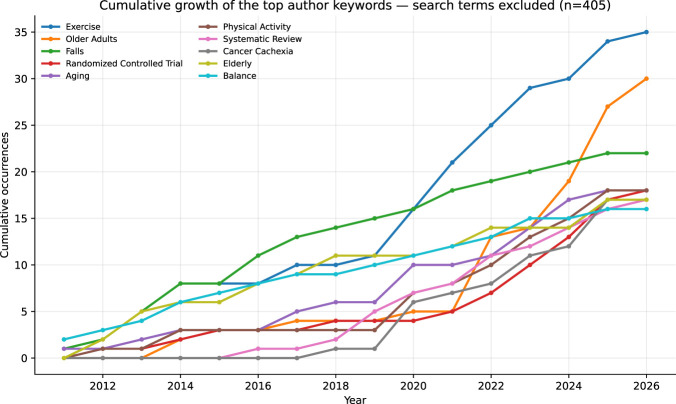
Cumulative growth of the top author keywords with search terms excluded.

**FIGURE 9:**
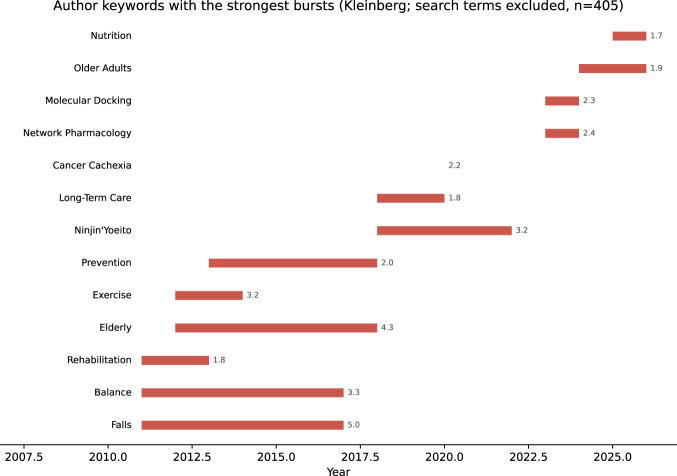
Author keywords with the strongest bursts (Kleinberg algorithm; search terms excluded).

**FIGURE 10:**
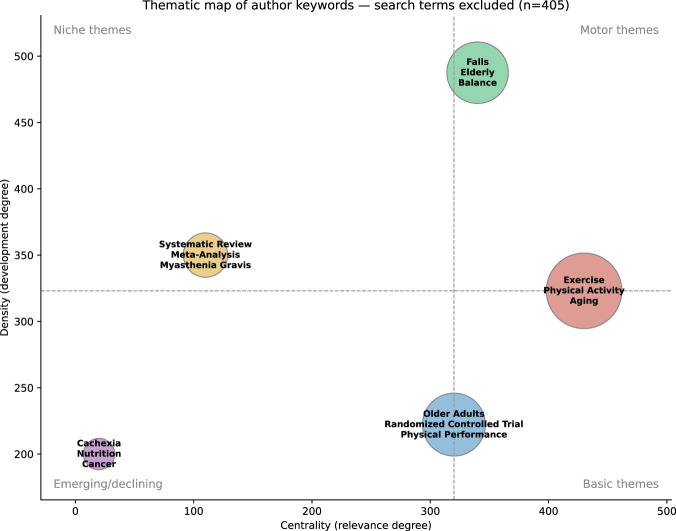
Thematic map of author keywords with search terms excluded.

Keyword growth trajectories for the top author keywords (search terms excluded) are shown in Figure 8: “cancer cachexia,” “network pharmacology,” “rehabilitation,” and “nutrition” show the steepest recent slopes. Burst detection (Figure 9) identified a clear temporal shift. The earliest and strongest bursts occurred in “falls” (2011–2017), “elderly” (2012–2018), “balance” (2011–2017), “exercise” (2012–2014), and “rehabilitation” (2011–2013), reflecting an early focus on fall prevention and functional performance in older adults. Mid-period bursts included “ninjin’yoeito” (2018–2022) and “cancer cachexia” (2020). The most recent bursts—“network pharmacology” and “molecular docking” (2023–2024), “older adults” (2024–2026), and “nutrition” (2025–2026)—signal a pronounced shift toward computational-mechanistic pharmacology and nutrition science. The thematic map (Figure 10) positioned *falls/elderly/balance* as a Motor Theme (high centrality and density); *exercise/aging/physical activity* and *older adults/randomized controlled trials/physical performance* as Basic Themes; *systematic review/meta-analysis* as a Niche Theme; and *cachexia/nutrition/cancer* as an Emerging or Declining Theme.

### 3.7 Reference co-citation and cluster analysis

**FIGURE 11:**
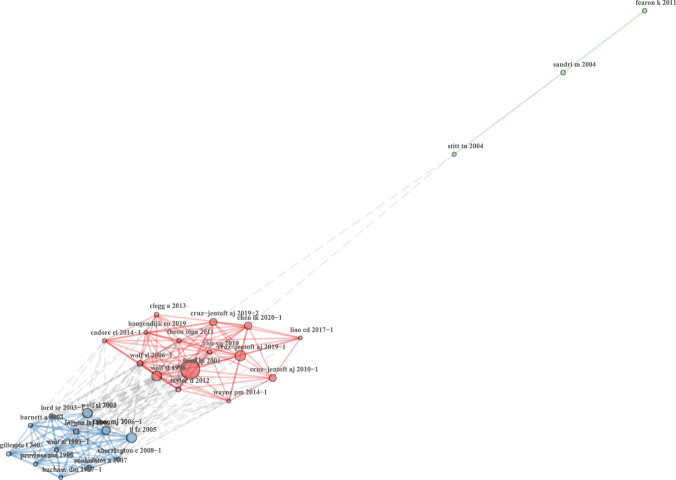
Reference co-citation network.

The reference co-citation network (Figure 11) revealed the intellectual base of the field. The largest node was Fried et al. (2001), the seminal paper defining the frailty phenotype (70 co-citations), which anchored the entire network, closely linked to the landmark frailty reviews of Clegg et al. (2013) and Hoogendijk et al. (2019). A prominent cluster of tai-chi fall-prevention trials—led by works of Wolf and colleagues and Li et al—formed the clinical-exercise pillar, consistent with the strong falls/balance signal in the keyword analysis. A distinct cluster centered on the sarcopenia consensus definitions—Cruz-Jentoft et al. (EWGSOP and EWGSOP2 (Cruz-Jentoft et al., 2019; Cruz-Jentoft et al., 2010)) and (AWGS 2019 Chen et al. (2020))—provided the diagnostic framework for sarcopenia studies. A further cluster linking cancer cachexia to geriatric muscle wasting was anchored by Fearon et al. (2011) and Baracos et al. (2018), indicating cross-fertilization between oncology-related muscle atrophy and geriatric sarcopenia research. Among the most globally cited documents in the dataset were Cadore et al. (2013) and Chou et al. (2012), both exercise-intervention syntheses.

### 3.8 Thematic evolution analysis

**FIGURE 12:**
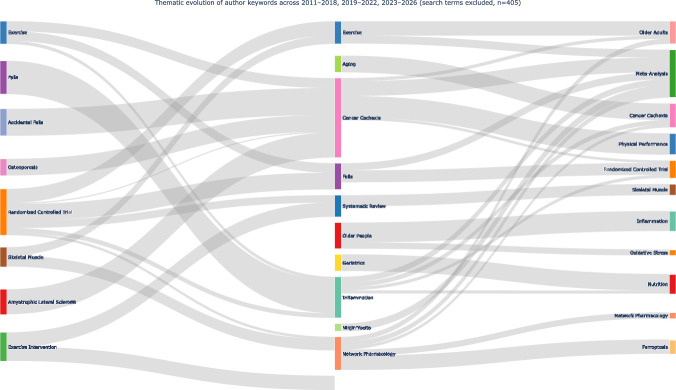
Thematic evolution of author keywords across 2011–2018, 2019–2022, and 2023–2026 (search terms excluded).

The thematic evolution analysis divided the period into three phases (Figure 12). During 2011–2018, the dominant themes were exercise, falls, accidental falls, osteoporosis, randomized controlled trials, and early muscle-atrophy work (including an amyotrophic lateral sclerosis strand), reflecting a focus on fall prevention and functional performance. In 2019–2022, the field diversified: cancer cachexia, aging, systematic review, inflammation, ninjin’yoeito, and network pharmacology emerged as major independent themes alongside continued falls research. In 2023–2026, a clear shift was evident: meta-analysis, cancer cachexia, physical performance, skeletal muscle, inflammation, oxidative stress, nutrition, network pharmacology, and ferroptosis became prominent—signaling maturation toward evidence synthesis and mechanistic/computational research. The evolution from clinical fall-prevention studies toward multi-modal, mechanistically grounded TCM research represents a maturation of the field and alignment with contemporary precision-medicine and integrative geriatric-care paradigms.

### 3.9 Cross-database validation

**FIGURE 13:**
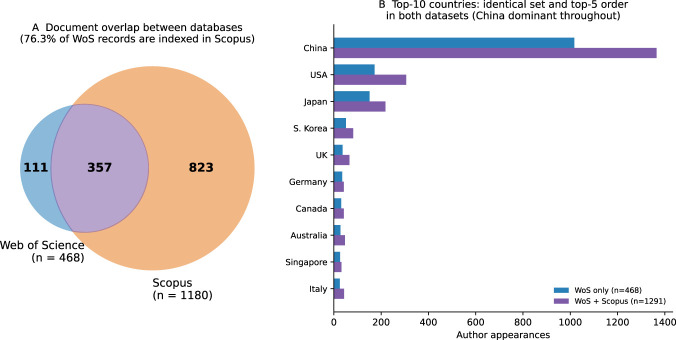
Cross-database validation against Scopus. **(A)** Overlap between the document-type-eligible Web of Science records (n = 468) and the equivalent Scopus retrieval (n = 1,180); 357 records — 76.3% of the Web of Science set—are common to both databases. **(B)** The ten most productive countries derived from Web of Science alone versus the merged Web of Science + Scopus corpus (n = 1,291); the set of countries and the top-five rank order are identical across datasets, with China dominant in both.

The equivalent Scopus search retrieved 1,180 articles and reviews. Of the 468 document-type-eligible Web of Science records, 357 (76.3%) were also indexed in Scopus (Figure 13A), indicating substantial agreement between the two databases on the core literature; merging the two sources with duplicates removed yielded 1,291 unique records. Critically, the knowledge structure was highly concordant across databases (Figure 13B; Supplementary Table S2). The ten most productive countries were identical in both datasets, with the top five (China, the United States, Japan, South Korea, and the United Kingdom) in the same rank order, and China overwhelmingly dominant throughout (1,017 author appearances in Web of Science versus 1,365 in the merged corpus). Eight of the ten leading journals were common to both datasets, and the dominant author-keyword themes (exercise, falls and balance, rehabilitation, older adults, and evidence synthesis [systematic review and meta-analysis]) were likewise consistent. Although Scopus retrieved a larger raw volume, reflecting its broader but less citation-standardized indexing, incorporating these additional records did not alter the structural findings but reinforced them. This cross-database concordance indicates that the knowledge map reported here reflects the research field itself rather than an artifact of single-database coverage.

## 4 Discussion

### 4.1 Overview of the research landscape

This is, to our knowledge, the first bibliometric analysis mapping TCM research specifically at the intersection of frailty and sarcopenia, covering 405 publications from 2011 to 2026. Annual output was modest through 2016, then accelerated sharply after 2019, peaking at 77 publications in 2025. The post-2019 acceleration coincides with the EWGSOP2 revised consensus (Cruz-Jentoft et al., 2019), which standardized sarcopenia diagnostic criteria and triggered a wave of intervention studies, alongside broader initiatives such as the WHO Decade of Healthy Ageing (2021–2030).

The geographic analysis revealed a striking concentration of output in China, which accounted for the overwhelming majority of publications—predictable given TCM’s origins and the scale of domestic research investment. More notable is the 17.78% international co-authorship rate. While Japan contributed significantly—driven primarily by Kampo research on formulas such as ninjin’yoeito—and the USA, South Korea, and European countries showed growing engagement, the limited cross-national collaboration means that findings from predominantly single-country studies will face questions about generalizability. Without harmonized protocols and multi-center trials, this gap will persist.

The 188 sources carrying this literature span geriatrics, pharmacology, nutrition, public health, and complementary medicine. That Phytomedicine and the Journal of Ethnopharmacology each carry a notable share indicates that TCM–sarcopenia research is increasingly treated as mainstream pharmacological science rather than a niche area of alternative medicine.

### 4.2 Hot topics and research frontiers

The keyword and thematic analyses—now free of the retrieval terms and therefore reflecting genuine research content—tell a consistent story about how this field has matured. The early period (2011–2018) was largely about clinical function: falls, balance, and exercise in older adults, with tai-chi fall-prevention trials forming the empirical core (as the co-citation structure confirms). The recent period (2023–2026) looks different: cancer cachexia, network pharmacology, molecular docking, oxidative stress, and nutrition now appear as independent research targets, meaning studies are increasingly designed around mechanisms and standardized outcomes rather than symptom management.

The sharpest recent signals are the bursts in network pharmacology and molecular docking (2023–2024) and in nutrition (2025–2026). The former reflects the rapid adoption of computational systems-pharmacology approaches to dissect the multi-target actions of herbal formulas; the latter reflects growing integration of nutritional science with TCM intervention design. On the thematic map, falls/elderly/balance occupies the Motor-Themes quadrant—well-developed and central—while cachexia/nutrition sits in the Emerging/Declining quadrant, indicating a young but rising area. There is still no consensus on which TCM intervention works best for sarcopenia specifically, by which outcome criteria, and in which patient subgroups; that remains the most concrete research gap this analysis identifies.

### 4.3 Mechanistic insights: bridging TCM theory and modern geroscience

The emergence of a mechanistic cluster—spanning oxidative stress, inflammation, skeletal muscle, and (in the most recent phase) ferroptosis—shows that TCM researchers are increasingly engaging with the biology of muscle aging rather than confining investigation to functional outcomes. Inflammaging—the chronic, low-grade systemic inflammation associated with aging (Franceschi et al., 2018)—drives muscle protein degradation through activation of the NF-κB pathway and the ubiquitin–proteasome system, in which the FoxO-regulated atrophy ligases characterized by Sandri et al. (2004) and Stitt et al. (2004) play a central role. Chinese herbal formulas with anti-inflammatory properties (e.g., those containing Astragalus membranaceus, Panax ginseng, and Curcuma longa) may modulate these pathways and attenuate muscle wasting. Oxidative stress is a related target: mitochondrial dysfunction and reactive-oxygen-species accumulation drive satellite-cell senescence and impair regeneration, and several herbal saponins and polyphenols have shown mitochondria-protective effects *in vitro*, though clinical translation remains limited.

The emerging gut–muscle axis is a particularly promising frontier. Gut-microbiota dysbiosis has been implicated in sarcopenia through altered amino-acid metabolism, systemic inflammation, and impaired nutrient absorption (Ticinesi et al., 2017). TCM herbal formulas, with well-documented modulatory effects on gut microbiota, may act through this pathway; the TCM framework of “Spleen governing muscles” (Pi Zhu Ji Rou) offers an intriguing conceptual parallel. Finally, the co-citation linkage between cancer-cachexia literature (Fearon et al., 2011) and geriatric sarcopenia—reinforced by the cancer-cachexia keyword cluster—points to shared pathways (myostatin signaling, autophagy dysregulation, satellite-cell exhaustion) where multi-target TCM formulas may have advantages over single-target drugs, though demonstrating this rigorously will require more specific mechanistic studies than currently exist.

### 4.4 Clinical translation and implementation challenges

The growth in meta-analyses and systematic reviews from 2019 onward signals that the field is generating enough primary literature to support evidence synthesis, but the quality of the primary studies constrains what the reviews can conclude. Many TCM clinical trials suffer from small sample sizes, inadequate blinding (particularly for non-pharmacological interventions such as tai chi and acupuncture), heterogeneous outcome measures, and short follow-up. Future trials should adopt rigorous designs aligned with CONSORT, use validated sarcopenia criteria (EWGSOP2 (Cruz-Jentoft et al., 2019) or AWGS 2019 (Chen et al., 2020)), and include patient-reported alongside objective functional measures.

Standardization is a related bottleneck: herbal formulas vary in composition, dose, preparation, and duration across studies, and exercise-based interventions (“tai chi” in one trial may differ substantially from another) are equally heterogeneous—agreed protocols are a prerequisite for meaningful meta-analysis. Safety in the context of polypharmacy also cannot be deferred, as herb–drug interactions in frail older adults are poorly characterized; systematic pharmacovigilance will be a prerequisite for any guideline recommendation.

### 4.5 Limitations

Several limitations should be acknowledged. First, the primary analysis was based on a single English-language database, the Web of Science Core Collection, chosen for the standardized, fully parsed cited-reference metadata that co-citation, bibliographic-coupling, and burst analyses require and that CNKI and most regional databases do not provide in a compatible form. To assess the impact of this choice, we cross-validated the findings against Scopus (Section 3.9): the two databases shared 76.3% of the Web of Science records, and the leading countries, journals, and keyword themes were concordant between Web of Science alone and the merged corpus, indicating that the knowledge structure is robust to database choice. Nevertheless, coverage bias remains a genuine limitation for a traditional-Chinese-medicine topic: research published in Chinese-language journals indexed only in CNKI, Wanfang, or VIP is captured by neither Web of Science nor Scopus, so the true domestic volume of research in this field is undercounted and the geographic and collaboration findings may be biased toward the international literature. This study is therefore best interpreted as a map of the international (English-language) knowledge structure of the field, and a complementary CNKI-based analysis of the Chinese-language literature is an important direction for future work (Section 4.6).

Second, the search relied on free-text terms because WoSCC does not offer MeSH/controlled vocabulary; although the strategy was deliberately sensitive and a formal relevance screening removed 63 off-topic records (Figure 1; Supplementary Table S1), residual imprecision cannot be entirely excluded. Third, bibliometric indicators such as citation counts are influenced by journal impact factor, publication age, and self-citation and do not directly reflect research quality. Fourth, keyword analysis depends on author-assigned keywords and Keywords Plus®, which carry semantic heterogeneity; data cleaning was performed, but residual inconsistency may remain. Finally, the cross-sectional search (14 March 2026) does not capture later-indexed publications, and bibliometric analysis cannot substitute for systematic reviews in evaluating the efficacy of specific interventions.

### 4.6 Future directions

Several priorities follow from these patterns. Most pressing is the need for adequately powered, multi-center randomized controlled trials using EWGSOP2 or AWGS 2019 criteria with follow-up long enough to detect clinically meaningful outcomes; tai chi, baduanjin, ninjin’yoeito, and a small number of herbal formulas with reasonable safety data are natural candidates. On the mechanistic side, the gut–muscle axis, inflammaging, and oxidative-stress pathways are where the most productive questions currently sit; the recent surge in network pharmacology and molecular docking indicates that multi-omics and systems-pharmacology approaches are already being marshalled to map which TCM compounds act on which nodes. The rehabilitation and nutrition trends suggest a near-term practical direction: integrating TCM mind-body exercise and nutritional strategies into existing prehabilitation and post-hospitalization programs. International collaboration will be necessary rather than optional if findings are to generalize beyond East Asian populations; an 18%-range co-authorship rate is not adequate for that purpose. Finally, future bibliometric work should incorporate additional databases—Scopus for the international literature and, crucially, CNKI for the Chinese-language literature—and consider natural-language processing of full-text content to reveal patterns that metadata-level keyword analysis cannot access.

## 5 Conclusion

This analysis mapped 405 publications on TCM for frailty and sarcopenia from 2011 to 2026 using the Web of Science Core Collection, retained through a documented four-phase screening. Output grew sharply after 2019, driven primarily by the EWGSOP2 diagnostic consensus and rising attention to healthy aging, and peaked at 77 publications in 2025. China accounts for the majority of publications, with the USA and Japan as secondary contributors; international co-authorship stands at 17.78%.

With retrieval terms removed from the keyword analyses, the knowledge structure resolves into genuine research streams: clinical exercise and fall-prevention research (the empirical core, anchored by tai-chi trials), randomized-controlled-trial outcomes of muscle strength and physical function, evidence synthesis, and a rising mechanistic/nutritional stream (cancer cachexia, network pharmacology, oxidative stress, inflammation, and nutrition). Thematic evolution shows a clear shift from clinical fall-prevention research (2011–2018) toward mechanistically grounded, computationally supported, and nutrition-oriented research (2023–2026). The intellectual base rests on the foundational frailty and sarcopenia papers (Cruz-Jentoft et al., 2019; Fried et al., 2001; Chen et al., 2020), with productive cross-disciplinary connections to cancer cachexia. The most pressing next steps are adequately powered multi-center trials with standardized criteria, mechanistic investigation of the gut–muscle axis and inflammaging pathways, integration of complementary databases (notably CNKI), and stronger international collaboration.
