# Supplementary material for: Traditional Chinese medicine for frailty and sarcopenia in aging populations: a bibliometric and visualized analysis of research trajectories and emerging frontiers
Source: Front Aging. 2026 Jul 31;7:1854710. doi: 10.3389/fragi.2026.1854710 (PMC13473409; doi:10.3389/fragi.2026.1854710)
Supplement: Supplementary file 1 [file Table1.docx]

# Supplementary Table S1. Records excluded during title/abstract relevance screening (n = 63)

The 468 document-type-eligible records were screened against the relevance criteria described in Methods (Section 2.2). Sixty-three records were excluded as not relevant to TCM interventions for frailty/sarcopenia, leaving 405 publications for the bibliometric analysis. Exclusion reasons: No frailty/sarcopenia/skeletal-muscle outcome (n = 36); TCM intervention not the subject of study (n = 18); Veterinary / non-human (n = 3); Phytochemistry / pharmacokinetics only (n = 2); Isolated drug-safety report (n = 2); Not a frailty/sarcopenia/aging focus (n = 2).

| No. | Year | Reason | Title |
| --- | --- | --- | --- |
| 1 | 2023 | Veterinary / non-human | Four Patterns of Canine Wei Syndrome Treated with Traditional Chinese Medicine |
| 2 | 2024 | No frailty/sarcopenia/skeletal-muscle outcome | Torsade de Pointes Caused by a Compound Licorice Tablet |
| 3 | 2011 | No frailty/sarcopenia/skeletal-muscle outcome | Berberine Improves Glucose Metabolism in Diabetic Rats by Inhibition of Hepatic Gluconeogenesis |
| 4 | 2021 | No frailty/sarcopenia/skeletal-muscle outcome | Novel Pharmacological Effects of Syringin on Anxiety Behavior and Autonomic Nervous System Activity |
| 5 | 2016 | No frailty/sarcopenia/skeletal-muscle outcome | Treatment of insulin resistance by acupuncture: a review of human and animal studies |
| 6 | 2022 | No frailty/sarcopenia/skeletal-muscle outcome | Gastrodin promotes the regeneration of peripheral nerves by regulating miR-497/BDNF axis |
| 7 | 2025 | No frailty/sarcopenia/skeletal-muscle outcome | Efficacy and safety of East Asian herbal medicine for treating depression in older adults: A systematic review and meta-analysis |
| 8 | 2026 | No frailty/sarcopenia/skeletal-muscle outcome | Utilization, user evaluation, and associated factors of Traditional Chinese Medicine techniques for insomnia symptoms among Chinese community-dwelling older adults: a cross-sectional study |
| 9 | 2023 | No frailty/sarcopenia/skeletal-muscle outcome | Traditional Chinese medicine for the prevention and treatment of presbycusis |
| 10 | 2014 | No frailty/sarcopenia/skeletal-muscle outcome | IMPROVEMENTS IN SLEEP AND HANDWRITING AFTER COMPLEMENTARY MEDICAL INTERVENTION USING ACUPUNCTURE, APPLIED KINESIOLOGY, AND RESPIRATORY EXERCISES IN A NINE-YEAR-OLD ADHD PATIENT ON METHYLPHENIDATE |
| 11 | 2024 | No frailty/sarcopenia/skeletal-muscle outcome | Alternative therapy for acute appendicitis with appendicolith using traditional Chinese medicine: A case report |
| 12 | 2018 | No frailty/sarcopenia/skeletal-muscle outcome | Everybody was Kung-Fu fighting-The beneficial effects of Tai Chi Qigong and self-defense Kung-Fu training on psychological and endocrine health in middle aged and older men |
| 13 | 2024 | TCM intervention not the subject of study | Frailty Predicts in-Hospital Death in Traumatic Brain Injury Patients: A Retrospective Cohort Study |
| 14 | 2024 | TCM intervention not the subject of study | Correlation between depression and adherence to respiratory function exercise in elderly frail patients with tuberculosis: A cross-sectional survey |
| 15 | 2024 | TCM intervention not the subject of study | Association of preoperative cognitive frailty with postoperative complications in older patients under general anesthesia: a prospective cohort study |
| 16 | 2025 | TCM intervention not the subject of study | Development and validation of an interpretable machine learning model for predicting low muscle mass in patients with rheumatoid arthritis: a multicenter study |
| 17 | 2024 | No frailty/sarcopenia/skeletal-muscle outcome | Pharmacological activity and clinical application analysis of traditional Chinese medicine ginger from the perspective of one source and multiple substances |
| 18 | 2025 | No frailty/sarcopenia/skeletal-muscle outcome | A multi-center, randomized, double-blind, placebo-controlled trial to evaluate the efficacy and safety of Fuzheng Yangxin Granule in treating heart failure with preserved ejection fraction (Qi-Yin deficiency and blood stasis syndrome): study protocol |
| 19 | 2025 | TCM intervention not the subject of study | Vitamin D and cancer-related fatigue in elderly patients: mechanisms and therapeutic insights-a narrative review |
| 20 | 2024 | No frailty/sarcopenia/skeletal-muscle outcome | Exploration on pharmacological mechanisms of YZP against neuropathic pain via inhibiting spinal inflammation and the rationality of its compatibility |
| 21 | 2025 | TCM intervention not the subject of study | Correlation between the severity of lumbar spinal stenosis and lumbar paraspinal muscle atrophy |
| 22 | 2012 | No frailty/sarcopenia/skeletal-muscle outcome | Psychological effects of Tai Chi Chuan |
| 23 | 2024 | No frailty/sarcopenia/skeletal-muscle outcome | Acute effects of different Tai Chi practice protocols on cardiac autonomic modulation |
| 24 | 2024 | No frailty/sarcopenia/skeletal-muscle outcome | Tetrahydroberberine Prevents Ovariectomy-Induced Bone Loss by Inhibiting RANKL-Induced Osteoclastogenesis and Promoting Osteoclast Apoptosis |
| 25 | 2025 | TCM intervention not the subject of study | Anemia, as an adverse event during chemoradiotherapy for head and neck cancer, contributes to a reduction in swallowing muscle mass and leads to dysphagia |
| 26 | 2025 | No frailty/sarcopenia/skeletal-muscle outcome | Lycium barbarum Polysaccharide Ameliorates Hindlimb Unloading-Induced Bone Loss by Targeting Noggin |
| 27 | 2023 | No frailty/sarcopenia/skeletal-muscle outcome | Impact of wrist-ankle acupuncture on propofol dosage under the dual monitoring of density spectrum array and anesthesia consciousness index in elderly patients undergoing urologic surgery: a sham-controlled randomized clinical trial |
| 28 | 2023 | No frailty/sarcopenia/skeletal-muscle outcome | The Role of Rehabilitation in the Treatment of Constipation in Oncological Patients |
| 29 | 2025 | TCM intervention not the subject of study | Cachexia in Pancreatic Cancer: New Insights to Impact Quality of Life and Survival |
| 30 | 2020 | Veterinary / non-human | ASIAN ELEPHANT (ELEPHAS MAXIMUS) SUFFERING FROM LIGHTNING STRIKE SUCCESSFULLY TREATED BY INTEGRATIVE VETERINARY MEDICINE |
| 31 | 2025 | TCM intervention not the subject of study | Prognostic Value of Frailty in Patients With Takotsubo Cardiomyopathy |
| 32 | 2016 | TCM intervention not the subject of study | Current Trends in Geriatric Emergency Medicine |
| 33 | 2025 | TCM intervention not the subject of study | Assessment of bidirectional relationships between sleep traits and frailty: A bidirectional Mendelian randomization study |
| 34 | 2024 | Veterinary / non-human | Epidemiology of Canine Wei Syndrome and Its Hemorheology Characteristics |
| 35 | 2021 | Phytochemistry / pharmacokinetics only | Pharmacokinetic study of Ninjin’yoeito: Absorption and brain distribution of Ninjin’yoeito ingredients in mice |
| 36 | 2024 | Phytochemistry / pharmacokinetics only | A systematic review on polysaccharides from Morinda officinalis How: Advances in the preparation, structural characterization and pharmacological activities |
| 37 | 2020 | Isolated drug-safety report | Risk Factors for Pseudoaldosteronism with Yokukansan Use: Analysis Using the Japanese Adverse Drug Report (JADER) Database |
| 38 | 2025 | No frailty/sarcopenia/skeletal-muscle outcome | Knowledge, attitudes, and practices regarding intrinsic capacity in the elderly population: A cross-sectional study in Shanghai, China |
| 39 | 2024 | No frailty/sarcopenia/skeletal-muscle outcome | Mechanism of Acupuncture in Treating Obesity: Advances and Prospects |
| 40 | 2023 | No frailty/sarcopenia/skeletal-muscle outcome | Transcriptomic analysis the mechanisms of anti-osteoporosis of desert-living Cistanche herb in ovariectomized rats of postmenopausal osteoporosis |
| 41 | 2019 | No frailty/sarcopenia/skeletal-muscle outcome | Anti-Osteoporotic Activity of an Edible Traditional Chinese Medicine Cistanche deserticola on Bone Metabolism of Ovariectomized Rats Through RANKL/RANK/TRAF6-Mediated Signaling Pathways |
| 42 | 2022 | No frailty/sarcopenia/skeletal-muscle outcome | Integrated Care Components in Transitional Care Models from Hospital to Home for Frail Older Adults: A Systematic Review |
| 43 | 2022 | No frailty/sarcopenia/skeletal-muscle outcome | Ninjinyoeito ameliorated cigarette smoke extract-induced apoptosis and inflammation through JNK signaling inhibition in human lung fibroblasts |
| 44 | 2025 | TCM intervention not the subject of study | Unveiling the relation between swallowing muscle mass and skeletal muscle mass in head and neck cancer patients |
| 45 | 2025 | Not a frailty/sarcopenia/aging focus | Blood flow restriction training combined with muscle energy technology for upper limb functional recovery in poststroke spasticity: a randomised controlled trial protocol at a tertiary hospital in Jiaxing, China |
| 46 | 2025 | TCM intervention not the subject of study | Phenotypic and functional dysregulations of CD8+T Cells in myasthenia gravis |
| 47 | 2020 | Not a frailty/sarcopenia/aging focus | Rehabilitation Interventions for Shoulder Dysfunction in Patients With Head and Neck Cancer: Systematic Review and Meta-Analysis |
| 48 | 2019 | No frailty/sarcopenia/skeletal-muscle outcome | Spot the silent sufferers: A call for clinical diagnostic criteria for solar and nutritional osteomalacia |
| 49 | 2023 | TCM intervention not the subject of study | Conditioned Media from Head and Neck Cancer Cell Lines and Serum Samples from Head and Neck Cancer Patients Drive Catabolic Pathways in Cultured Muscle Cells |
| 50 | 2015 | No frailty/sarcopenia/skeletal-muscle outcome | Pain management in residential aged care facilities |
| 51 | 2023 | TCM intervention not the subject of study | Combining a Frailty Index Based on Laboratory Data and Pneumonia Severity Assessments to Predict In-Hospital Outcomes in Older Adults with Community-Acquired Pneumonia |
| 52 | 2024 | TCM intervention not the subject of study | Rare complication of rheumatoid arthritis: Charcot Neuro-osteoarthropathy |
| 53 | 2025 | No frailty/sarcopenia/skeletal-muscle outcome | Caregiver beliefs about older adult falls from a nationally representative U. S. sample 2022 |
| 54 | 2023 | Isolated drug-safety report | Side-effects of hyperthermic intraperitoneal chemotherapy in patients with gastrointestinal cancers |
| 55 | 2016 | TCM intervention not the subject of study | Multiple sclerosis: New insights and trends |
| 56 | 2017 | TCM intervention not the subject of study | Multiple sclerosis: New insights and trends |
| 57 | 2020 | No frailty/sarcopenia/skeletal-muscle outcome | Nutritional rickets & osteomalacia: A practical approach to management |
| 58 | 2024 | No frailty/sarcopenia/skeletal-muscle outcome | Effect of prophylactic infusion of norepinephrine on the prevention of hypotension during vertebroplasty: a randomized clinical trial |
| 59 | 2020 | No frailty/sarcopenia/skeletal-muscle outcome | Components of the transitional care model (TCM) to reduce readmission in geriatric patients: a systematic review |
| 60 | 2020 | No frailty/sarcopenia/skeletal-muscle outcome | Can voucher scheme enhance primary care provision for older adults: cross-sectional study in Hong Kong |
| 61 | 2023 | No frailty/sarcopenia/skeletal-muscle outcome | Phytochemicals and Vitamin D for a Healthy Life and Prevention of Diseases |
| 62 | 2024 | No frailty/sarcopenia/skeletal-muscle outcome | The development and flux of the University of Minnesota Survivorship Program: progress, challenges, and opportunities |
| 63 | 2023 | No frailty/sarcopenia/skeletal-muscle outcome | Performance of Early Warning Scoring Systems Regarding Adverse Events of Unanticipated Clinical Deterioration in Complementary and Alternative Medicine Hospitals |
