# Supplementary material for: Traditional Chinese medicine for frailty and sarcopenia in aging populations: a bibliometric and visualized analysis of research trajectories and emerging frontiers
Source: Front Aging. 2026 Jul 31;7:1854710. doi: 10.3389/fragi.2026.1854710 (PMC13473409; doi:10.3389/fragi.2026.1854710)
Supplement: Supplementary file 2 [file Table2.docx]

# Supplementary Table S2. Cross-database validation: concordance of the leading entities between Web of Science alone and the merged Web of Science + Scopus corpus

The equivalent search in Scopus retrieved 1,180 articles/reviews; 357 (76.3%) of the 468 document-type-eligible Web of Science records were also indexed in Scopus, and merging the two databases (duplicates removed) yielded a union of 1,291 records. The tables below compare the ten leading countries, journals, and author keywords (search terms excluded) derived from Web of Science alone versus the merged corpus. Ranks are highly concordant across databases, indicating that the knowledge structure reported in the main analysis is robust to the choice of database rather than an artifact of single-database coverage.

## (a) Most productive countries (author appearances)

| Rank | Web of Science only (n = 468) | Web of Science + Scopus (n = 1,291) |
| --- | --- | --- |
| 1 | China (1,017) | China (1,365) |
| 2 | USA (172) | USA (306) |
| 3 | Japan (151) | Japan (218) |
| 4 | South Korea (51) | South Korea (82) |
| 5 | United Kingdom (37) | United Kingdom (66) |
| 6 | Germany (35) | Australia (47) |
| 7 | Canada (31) | Italy (43) |
| 8 | Australia (28) | Canada (42) |
| 9 | Singapore (26) | Germany (42) |
| 10 | Italy (25) | Singapore (32) |

*The ten most productive countries are identical in both datasets, with the top five in the same rank order; China is overwhelmingly dominant in both.*

## (b) Most prolific journals

| Rank | Web of Science only | Web of Science + Scopus |
| --- | --- | --- |
| 1 | Medicine (20) | Journal of Ethnopharmacology (29) |
| 2 | BMC Geriatrics (15) | Frontiers in Pharmacology (24) |
| 3 | Journal of Ethnopharmacology (15) | Medicine (21) |
| 4 | Frontiers in Pharmacology (14) | BMC Geriatrics (16) |
| 5 | Frontiers in Nutrition (12) | Phytomedicine (16) |
| 6 | Frontiers in Public Health (11) | BMJ Open (15) |
| 7 | BMJ Open (10) | Chinese Journal of Tissue Engineering Research (13) |
| 8 | Evidence-Based Complementary and Alternative Medicine (9) | Evidence-Based Complementary and Alternative Medicine (13) |
| 9 | Phytomedicine (9) | Frontiers in Nutrition (12) |
| 10 | BMC Complementary Medicine and Therapies (8) | Frontiers in Public Health (11) |

*Eight of the ten leading journals are common to both datasets. (In the merged set, “Medicine” and “Medicine (United States)” — the same journal under different database naming conventions — appear separately; combined they remain the leading multidisciplinary outlet.) Scopus adds Chinese-indexed outlets such as the Chinese Journal of Tissue Engineering Research.*

## (c) Most frequent author keywords (search terms excluded)

| Rank | Web of Science only | Web of Science + Scopus |
| --- | --- | --- |
| 1 | exercise (35) | exercise (58) |
| 2 | older adults (32) | older adults (40) |
| 3 | falls (22) | rehabilitation (40) |
| 4 | systematic review (20) | aging (36) |
| 5 | aging (19) | systematic review (33) |
| 6 | randomized controlled trial (19) | falls (32) |
| 7 | physical activity (18) | elderly (31) |
| 8 | cancer cachexia (17) | balance (27) |
| 9 | elderly (17) | quality of life (27) |
| 10 | balance (16) | randomized controlled trial (26) |

*The dominant keyword themes — exercise, falls/balance, rehabilitation, older adults, and evidence synthesis (systematic review / meta-analysis) — are the same in both datasets, confirming that the thematic structure is stable across databases.*
